# Clinical benefit of additional whole-exome sequencing over panel sequencing in an all-comer real-world molecular tumor board

**DOI:** 10.1016/j.esmoop.2025.105894

**Published:** 2025-11-25

**Authors:** E. Krieghoff-Henning, T. Michaeli, T. Boch, J. Kirchhof, V. Haselmann, M. Neumaier, W.-K. Hofmann, J. Betge, M. Ebert, A. Teufel, V. Ast, C. Sauer, C. Cotarelo, R. Lozynskyy, M. Janning, F. Marmé, M. Sütterlin, A. Streuer, F. Siegel, C. Brochhausen, M. Collienne, D. Nowak, S. Loges

**Affiliations:** 1 Department of Personalized Oncology, University Hospital Mannheim, Heidelberg University, Mannheim, Germany; 2 Division of Personalized Medical Oncology, German Cancer Research Center (DKFZ), Heidelberg, Germany; 3 DKFZ Hector Cancer Institute at the University Medical Center Mannheim, Mannheim, Germany; 4 Division of Translational Medical Oncology, German Cancer Research Center (DKFZ), Heidelberg, Germany; 5 National Center for Tumor Diseases (NCT), NCT Heidelberg, a partnership between DKFZ and Heidelberg University Hospital, Heidelberg, Germany; 6 Department of Hematology and Oncology, Medical Faculty Mannheim of the Heidelberg University, Mannheim, Germany; 7 Heidelberg Institute for Stem Cell Technology and Experimental Medicine (HI-STEM gGmbH), Heidelberg, Germany; 8 Division of Stem Cells and Cancer, German Cancer Research Center (DKFZ) and DKFZ-ZMBH Alliance, Heidelberg, Germany; 9 Mannheim Cancer Center (MCC), Center for Clinical Trials, Medical Faculty Mannheim of the Heidelberg University, Mannheim, Germany; 10 Institute for Clinical Chemistry, University Medical Centre Mannheim, Medical Faculty Mannheim, Heidelberg University, Mannheim, Germany; 11 Junior Clinical Cooperation Unit Translational Gastrointestinal Oncology and Preclinical Models, German Cancer Research Center (DKFZ), Heidelberg, Germany; 12 Department of Medicine II, Medical Faculty at Mannheim, University of Heidelberg, Mannheim, Germany; 13 Division of Hepatology, Division of Bioinformatics, Department of Medicine II, Medical Faculty Mannheim, Heidelberg University, Mannheim, Germany; 14 Clinical Cooperation Unit Healthy Metabolism, Center for Preventive Medicine and Digital Health Baden-Württemberg (CPDBW), Medical Faculty Mannheim, Heidelberg University, Mannheim, Germany; 15 Department of Pathology, University Medical Centre Mannheim, Heidelberg University, Mannheim, Germany; 16 German Center for Lung Research (DZL), German Cancer Consortium (DKTK), Heidelberg, Germany; 17 Department of Gynecology and Obstetrics, University Medical Centre Mannheim, Medical Faculty Mannheim, Heidelberg University, Mannheim, Germany; 18 Department of Biomedical Informatics, Mannheim Institute for Intelligent Systems in Medicine (MIISM), Medical Faculty Mannheim, Heidelberg University, Mannheim, Germany

**Keywords:** molecular tumor board, whole-exome sequencing, WES, panel sequencing, biomarkers

## Abstract

**Background:**

Panel sequencing, whole-exome sequencing (WES) and whole-genome sequencing (WGS) often uncover therapeutic targets for cancer patients. However, it is still largely unclear to what extent patients directly benefit from broader analyses over panel sequencing alone.

**Materials and methods:**

We analyzed the molecular findings and recommendations issued by our molecular tumor board (MTB) in a cohort of patients who had received both a well-established diagnostic panel of medium size (up to 203 genes) and in-house WES, focusing on the number of recommendations that were issued on the basis of WES results only.

**Results:**

Our cohort consisted of 38 patients with advanced cancers, of whom about two-thirds had common and one-third had rare cancers. They received a total of 45 (range 0-4) treatment recommendations overall, of which 29 had a clinical level of evidence (LoE) and/or entailed a feasible study enrollment and were thus considered highly actionable. Sixteen recommendations, of which seven were highly actionable, were issued only on the basis of WES results (five own, two previous WES). Three out of those seven recommendations and one additional recommendation based on a previous large panel were related to complex molecular biomarkers such as homologous recombination deficiency or high tumor mutational burden, with poly (ADP-ribose) polymerase inhibitors or checkpoint inhibitors as recommended treatment. As expected, a higher proportion of the WES-only recommendations (63% versus 42% of recommendations overall) were based on non-clinical LoEs. One of eight recommendations implemented so far was based on biomarkers derived by WES only.

**Conclusions:**

In our MTB, WES enabled some additional clinically highly actionable recommendations for selected patients, suggesting that some patients do benefit from additional WES. These recommendations were often related to complex biomarkers, which may in principle also be derived from larger panels. These findings should be re-investigated prospectively in larger cohorts.

## Highlights

• We assessed the benefit of adding WES to panel sequencing in an all-comer MTB.
• Additional WES led to 16 further MTB recommendations, of which 7 were clinically highly actionable.
• In three cases, biomarkers underlying these recommendations were molecular patterns (TMB-high and BRCAness).
• Thus, WES had a distinct, but limited benefit in our all-comer MTB.

## Introduction

In precision oncology, treatment recommendations for individual cancer patients are issued on the basis of—often extensive—biomarker analyses, a strategy that has the potential to improve patient survival.^1^, ^2^, ^3^

For some biomarker/therapy combinations, a clear link between biomarkers and response to a particular treatment has already been established in clinical trials, leading to regulatory approval. However, for many, usually rare, cancer entities, this is not yet the case, and thus treatment options are often very limited.^4^^,^^5^ Moreover, even for well-studied cancers at later stages, at some point standard therapy options are eventually exhausted. To offer these patients further treatment options, broad genomic biomarker analyses are conducted to discover potential additional therapy targets and/or to facilitate participation in clinical trials.

These genomic biomarker analyses can span wide ranges from the analysis of a handful of genes or proteins over panels of several hundred genes to whole-exome sequencing (WES) or even whole-genome sequencing (WGS).^1^, ^2^, ^3^ The processed results of these analyses are then discussed and used to derive treatment recommendations in multidisciplinary molecular tumor boards (MTBs).^6^ First studies have shown that certain subgroups such as patients with rare cancers, but also patients with common cancers and many known therapy targets, may benefit from extensive tumor characterization for therapy selection, with a focus on the palliative setting.^4^^,^^7^, ^8^, ^9^, ^10^

By now, evidence on the impact of sequence variations, rearrangements or amplifications of specific cancer-related genes has grown so much that many of the most common, recurring changes can be detected using gene panels of up to a few hundred cancer-associated genes. In contrast, rarer and/or more complex genomic changes, such as tumor mutational burden (TMB) or a homologous recombination deficiency (HRD), are not well detectable with many of the routinely used ‛targeted’ gene panels of up to ∼200 genes in size, and alterations in uncommonly altered genes may also be missed.^11^, ^12^, ^13^, ^14^ Carrying out WES or WGS yields substantially more information, including information on germline alterations.^14^^,^^15^ However, it is more costly and time-consuming, requires more tissue and a higher sample quality and increases the complexity of the results and thereby their interpretability.^14^ Also, standardization remains an issue for WES or WGS pipelines.^16^ As the frequently occurring highly actionable mutations are included in established targeted panels, the question arises for which cancer patients and in which setting WES is likely to provide an additional benefit.^14^ Of note, even though individual cancer patients may not benefit directly from WES of their tumor genome, findings from broader and untargeted analyses have the potential to generate knowledge that may enable the development of new therapies for future cancer patients, provided that the data are systematically collected and investigated.

So far, many studies have attempted to determine the benefit of either targeted panel analysis or more extensive analyses such as WES/WGS. In contrast, few studies have analyzed systematically which patients might benefit directly from a very broad biomarker analysis such as WES compared with gene panel analysis alone, with or without additional messenger RNA (mRNA) sequencing. They indicate a distinct but limited potential benefit.^15^^,^^17^, ^18^, ^19^ In this study, we report our experience regarding a diverse all-comer cohort of 38 patients from our MTB for whom both panel sequencing and WES analysis were carried out.

## Materials and methods

### Study design, patients and selection criteria

We retrospectively identified and included all patients from the University Medical Center Mannheim (UMM), Mannheim, Germany, who were discussed in our MTB and received at least two different biomarker analyses, i.e. tissue panel analysis and WES (different pipelines), from 2020 to 2023. WES was mostly carried out for young patients or cases with specific clinical hypotheses. In cases where only one set of recommendations was available (for the MTB discussion based on either the panel sequencing or WES), WES as the basis for the respective recommendations was inferred from the other recommendations or directly from the results of the analysis (e.g. no mutations detected).

This study was conducted in accordance with the principles of the Declaration of Helsinki. All 38 participating patients provided written informed consent for the storage and analysis of their biomaterials and data for usage in the MTB and further biomedical research. The study was approved by the Ethics Committee II of Heidelberg University (2025-836).

### Molecular analyses

We used three tissue analysis panels from the Oncomine platform (https://www.oncomine.com/, ThermoFisher Scientific, Waltham, MA). Early MTB patient samples were analyzed with the Oncomine Focus assay (OFA, 52 genes) up until the end of 2021; from July 2020 onward we used the Oncomine Comprehensive v3 (OCA, 161 genes). Thus, the use of OFA and OCA overlapped. From April 2022, we also employed the Oncomine Childhood Cancer Research Panel (203 genes), as appropriate with respect to patient age and/or cancer entity. WES was initially carried out using the Illumina DNA Prep Kit (Illumina, San Diego, CA) in combination with the xGEN™ Hybridization Capture of DNA Libraries Kit (IDT, Coralville, IA) for target enrichment via hybrid capture and downstream bioinformatics analysis by an in-house pipeline.^20^ Since mid-2023 the target region of the bioinformatics pipeline was reduced to a virtual panel including all genes with known somatic variations that are potentially druggable and was further modified as prerequisite to successfully obtain an accreditation according to DIN EN ISO 15189. This accredited bioinformatics pipeline encompasses 591 genes and is therefore rather a large and comprehensive panel than a complete WES, but in contrast to the Oncomine panels, it contains a germline control and is easily extendable within the flexible accreditation.

For a comparison of the Oncomine panels and of the accredited pipeline used in this study, see Supplementary Table S1, available at https://doi.org/10.1016/j.esmoop.2025.105894. Note that the panels also encompass targeted mRNA sequencing for selected genes and allow for the detection of selected gene fusions and copy number variants. The in-house non-accredited pipeline includes TMB analysis.

### Metrics and statistical analyses

The primary goal of the study was to determine the number of ‛highly actionable’ recommendations that were issued on the basis of the WES analyses and explicitly not on the basis of the panel sequencing. We define highly actionable recommendations as recommendations with a clinical level of evidence (LoE) or recommendations for inclusion of the respective patient in a defined clinical study. LoEs were assigned to the observed alterations according to the German Cancer Research Center (DKFZ)/National Center for Tumor Diseases (NCT)/German Cancer Consortium (DKTK) MASTER LoEs, with m2C as the lowest LoE with a clinical level, namely case reports with the same biomarker but another cancer entity.^2^^,^^4^^,^^21^^,^^22^ Targets with a preclinical LoE (LoE m3 or m4) are considered less clinically actionable. This system is used by the major precision oncology programs in Germany and differs slightly from the European Society for Medical Oncology (ESMO) scale of clinical actionability.^23^ Additionally, ESMO Scale for Clinical Actionability of molecular Targets (ESCAT) tiers were assigned retrospectively. All WES-detected alterations were screened individually by biomedical experts for their potential prognostic or predictive relevance. Secondary goals were the overall number of recommendations on all LoEs and on clinical LoEs only. For the accredited WES pipeline used from mid-2023 onward, only clinical-level recommendations were recorded in the MTB recommendations due to the complexity of the results and the recommendation policy. Statistical analyses were not carried out due to the limited number of patients included in the study and due to the heterogeneous molecular analyses. Calculations of median and average values were carried out in Excel.

## Results

### Usage of the MTB at UMM Mannheim overall in the study period

The MTB in our institution convenes in a biweekly rhythm. Between 2020 and 2023, 361 patients altogether, amounting to an average of 4.88 (range 0-12) patients per meeting, were discussed. More than half of all patients were registered by the Department of Personalized Oncology (*n* = 112) and by the Gynecology Clinic (*n* = 85), followed by the Gastroenterology and the Hematology and Oncology Departments (*n* = 48 and 43), respectively. MTB registrations increased from 39 patients in 2020 to 114 patients in 2023.

### The panel sequencing/WES comparison cohort

**Figure 1:**
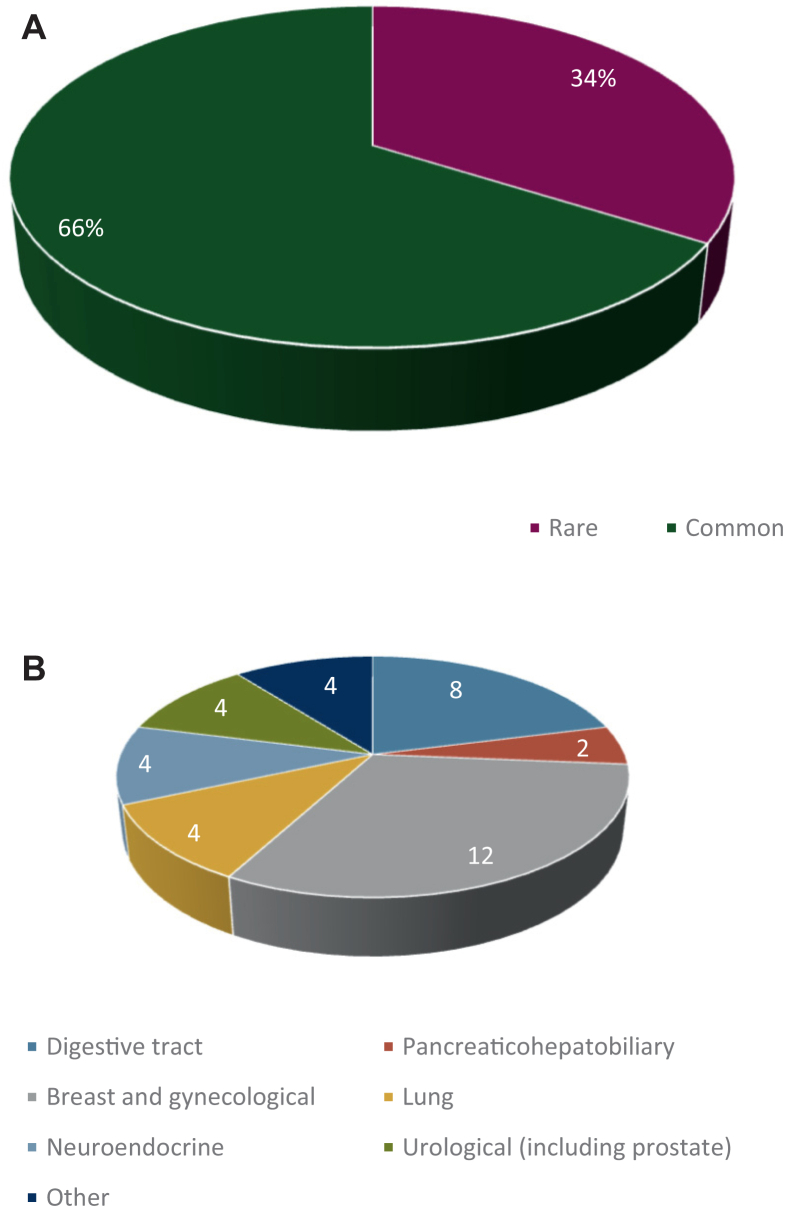
**Distribution of cancer entities. (**A) Common versus rare cancers. (B) Groups of cancer entities according to the organ system of origin.

Our cohort consisted of *n* = 38 patients (17 male, 21 female) of a median age at diagnosis of 53.5 years (range 22–79 years) with advanced cancer who were registered both for at least one panel sequencing and one WES analysis. The basic characteristics of this cohort are shown in Figure 1 and Supplementary Table S2, available at https://doi.org/10.1016/j.esmoop.2025.105894.

About two-thirds of the cancer entities relevant for the analyses (25 of 38) were common according to the RareCareNet list (http://rarecarenet.istitutotumori.mi.it/rarecarenet/index.php/cancerlist, mostly breast and ovarian cancer, gastrointestinal cancer and non-small-cell lung cancer), and about one-third (13 of 38) were rare (e.g. neuroendocrine tumors).

Many patients, especially patients with gynecological and breast cancers, were heavily pretreated (overall median of 2 therapy lines, average of 2.9, range 0-7 prior systemic therapies; gynecological + breast median of 3, average of 4.2, range 2-7, number unknown for four patients). WES was mostly requested/recommended immediately after the panel sequencing when results did not yield targetable molecular alterations with a clinical LoE of up to m2C.^2^^,^^4^^,^^21^ In two cases, WES was carried out before panel sequencing. In the panel sequencing analysis, we found a median of 2 and an average of 2.88 alterations (range 0-16) per patient. In the WES analysis, we found a median of 80 and an average of 191 (range 0-1993) alterations per patient. Importantly, this comparison does not include complex biomarkers such as HRD or TMB-high, which cannot be detected using the employed panels. The overlap between different analyses for the same patient was limited to 33 of 7187 WES-detected and 107 panel-detected individual alterations (Supplementary Figure S1, available at https://doi.org/10.1016/j.esmoop.2025.105894). This is in part due to the methodology: fusions (*n* = 4) could be detected using the mRNA part of the panels, but not using our WES pipeline, and amplifications (*n* = 19) could also only be detected by the panels. Also, germline variants are identified as variants in the panels, but not by our WES.

**Figure 2:**
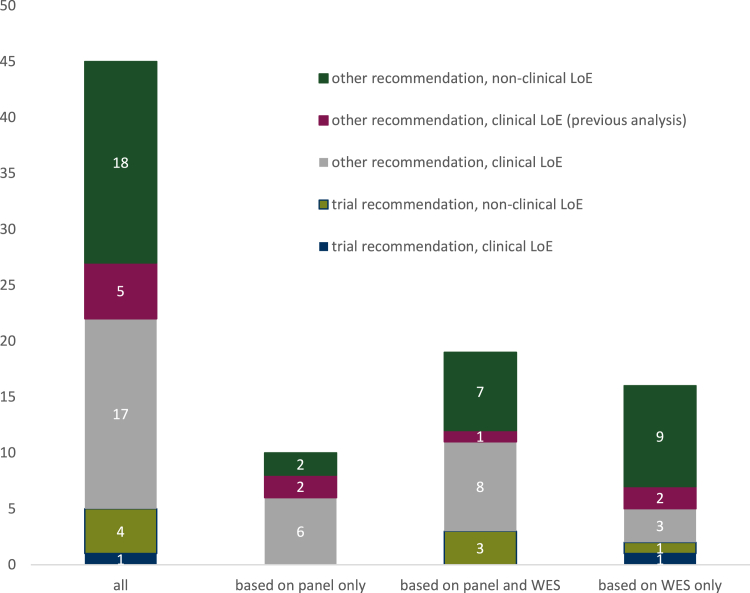
**Recommendations issued by the MTB**. As indicated, recommendations include both direct therapy recommendations and recommendations to participate in a clinical trial. A few recommendations issued by our MTB were based on previous panel or whole-exome sequencing. Non-clinical recommendations include DKFZ/NCT/DKTK MASTER LoEs m3 and m4. Clinical-level recommendations include LoEs up to level m2C. Note that only clinical-level recommendations were issued on the basis of the accredited pipeline that replaced the previous WES pipeline, so that potential recommendations with a preclinical LoE are not included for five patients. DKFZ, German Cancer Research Center; DKTK, German Cancer Consortium; LoE, level of evidence; MTB, molecular tumor board; NCT, National Center for Tumor Diseases; WES, whole-exome sequencing.

Altogether, 45 recommendations were issued by the MTB, of which 29 were highly actionable [clinical LoE according to the DKFZ/NCT/DKTK MASTER LoEs (*n* = 25) and/or clinical trial recommendation (*n* = 5), *n* = 1 with both a clinical trial recommendation and a clinical LoE] (Figure 2). All recommendations and biomarkers are summarized in Table 1. As expected, the proportion of recommendations with a preclinical LoE issued on the basis of WES results was numerically higher (10/16, 63% even including the accredited pipeline, where only alterations with clinical LoEs were called, versus 19/45 or 42% of recommendations overall). The MTB issued 7 biomarker-based highly actionable recommendations, i.e. recommendations with a clinical LoE or a potential study enrollment, out of 16 recommendations overall that were based on the WES (5 own, 2 previous WES) only, for seven individual patients (see Figure 2 and Table 1).

**Table 1:** Recommendations issued by the MTB

Patient	Recommendation	Biomarker(s)	LoE (MASTER)	ESCAT tier	Associated publication(s)	Based on
1	PD-(L)1 inhibitor	MUC19 variants (chr12:g.40941586C>A; chr12:g.40943917T>G)	m3	III-B	^24^	WES
	PD-(L)1 inhibitor	LRP1B variants (p. R2785K, p.D2786E)	m2C	III-B	^25^	WES
	PD-(L)1 inhibitor	RHEB variant (p.I163L)	m4	X	NCT03297606	WES
	PARP inhibitor	SLX4 variant (p.P931T)	m4	III-B	NCT03209401, NCT02677038, NCT03787680^26^	OCA/WES
2	Trametinib	NF1 variant (p.E1238)	m2C	III-B	27, 28, 29^,^^101^	OCA/WES
	Everolimus + eribulin	PTEN variants (p.Gln171∗ and p.Gln214∗)	m1A	III-B	30, 31, 32, 33, 34, 35, 36	OCA/WES
	Alpelisib + fulvestrant	PIK3CA variant (p.K111_I112delinsN)	m1B	I-A	^37^	OCA/WES
	Pembrolizumab or other checkpoint inhibitor	TMB-high	m2A	I-C	^38^	Previous WES
3	PARP inhibitor (if possible within study)	SMC1A variant (p.R747H)	m4	X	^39^^,^^40^	WES
4	Participation in TOPArt trial (NCT03127215, olaparib/trabectedin)	COSMIC BRCAness signature AC3	m2C^a^	III-B	^41^^,^^42^	WES
5	Checkpoint inhibitor	EPHA7 variant (p.P606T)	m4	III-B	^43^	WES
6	PARP inhibitor	CHEK2 variant (p.T367Mfs∗15)	m2A	III-B	^44^^,^^45^	OCA
8	Erdafitinib	FGFR3 variant (p.S249C)	m1A	I-A	^46^^,^^47^	OFA/WES (FGFR3 variant), OFA (FGFR2 amp)
9	Lenvatinib or other FGFR inhibitors	FGFR1 amplification	m2C^a^	III-A	^48^	OFA
10	CDK4/6 inhibitor	CCND1 amplification	m2C^a^	III-B	49, 50, 51	Previous panel
11	ERBB inhibitor, e.g. afatinib	ERBB3 variant (p.D297Y)	m2C	III-B	52, 53, 54, 55	OFA/previous WES
	Checkpoint inhibitor	TMB-high	m1A^a^	I-A	56, 57, 58, 59	Previous WES
17	Trametinib	NF1 variant (p.Q1360X)	m2C	III-B	^29^^,^^60^, ^61^, ^62^^,^^102^	WES
19	PARP inhibitor	RAD51C variant (p.R212C)	m4	IV-B	63, 64, 65	OFA, WES
20	CDK4/6 inhibitor, e.g. palbociclib	CTNNB1 variant (p.D32N)	m3	IV-A	66, 67, 68	OFA/WES
21	Alpelisib	PIK3CA variant (p.Q546K)	m2A	III-A	^37^	OCA/WES
22	PARP inhibitor	BLM variant (BLM P287Sfs∗4), RAD54L variant (p.E159X)	m4	IV-B	^69^	WES
	Participation in clinical trial with M4344 (NCT02278250) or temsirolimus)	ARID1A variant (D1850Gfs∗4)	m4	III-B	^70^	WES
	PARP inhibitor	RAD54L variant (p.E159X)	m3	IV-B	^63^	WES
24	Participation in trial with SOS1 inhibitor (NCT04111458)	KRAS variant (p.G12D)	m4	IV-A	^71^	OCA/WES
	Vismodegib	PTCH1 variant (p.Q1300H∗fs25)	m4		^72^	OCA
	TAS0612, within trial (NCT04586270)	KRAS variant (p.G12D)	m3	IV	^73^	OCA, WES
26	Checkpoint inhibitor, e.g. within IMP-150 treatment (carboplatin, paclitaxel, bevacizumab, atezolizumab)	TMB-high	m1A^a^ (checkpoint inhibitor)	I-C	https://www.fda.gov/drugs/drug-approvals-and-databases/fda-approves-pembrolizumab-adults-and-children-tmb-h-solid-tumors	Previous (large) panel
	PIK3CA inhibitor, e.g. alpelisib	PIK3CA variant (H1047R)	m2A	III-A	^37^	OCA, WES
	Osimertinib + PIK3CA inhibitor	EGFR variant (p.E479_A483del), PIK3CA variant (p.H1047R)	m3 (EGFR variant), m2A (PIK3CA variant)	IV (EGFR variant), III-A (PIK3CA variant)	^74^	OCA, WES
27	Participation in clinical trial with SOS1 inhibitor (NCT04111458)	KRAS variant (p.G12V)	m4^a^	IV (or III-B)	NCT04835714, NCT04111458	OCA, WES
28	AKT inhibitor, e.g. capivasertib, ipatasertib	AKT2 amplification	m4	IV	^75^	OCA
29	Alpelisib + fulvestrant	PIK3CA variant (p.G1049R)	m1A	I-A	^37^	OCA/WES
	CDK4/6 inhibitor + everolimus	CDK6 amplification, concomitant FGFR1 amplification	m1C	II-A (everolimus)	76, 77, 78	OCA
	FGFR inhibitor, e.g. erdafitinib + CDK4/6 inhibitor	CDK6 amplification, concomitant FGFR1 amplification	m1A	I-B	79, 80, 81	OCA
	Pazopanib	FGFR1 amplification	m1C	II-A or IV (imperfect fit)	^82^	OCA
30	Sotorasib	KRAS p.G12C	m1A^a^	I-B	^71^^,^^83^	OCA/WES
	CDK4/6 inhibitor, e.g. abemaciclib	CDKN2A variant (p.G72E)	m3	III-B	^84^^,^^103^	WES
	CDK4/6 inhibitor, e.g. abemaciclib	Pim1 variant (p.Q128L)	m3	IV	^84^^,^^103^	WES
	PARP inhibitor, e.g. talazoparib	STAG2 variant (p.P1217Q)	m3	IV	^85^	WES
33	PARP inhibitor, e.g. talazoparib	CHEK2 variant (c.1116_1117delCAinsTG)	m2A	III-A	^86^	WES
35	Pembrolizumab + lenvatinib	Amplification of FGF3, FGF19, FGFR1	m1A	IV	87, 88, 89, 90, 91, 92, 93	OCA
36	Platinum-based chemotherapy	BAP1 variant (p.C91G)	m2B^a^	III-A	94, 95, 96, 97, 98	OCA/WES (ap)
	PARP inhibitor	BAP1 variant (p.C91G)	m2C	III-A (imperfect fit)	^99^	OCA/WES (ap)
	EZH2 inhibitor	BAP1 variant (p.C91G)	m2A	III-A	^100^	OCA/WES (ap)

Note that TMB-h is not detectable in our panels, but was detected by WGS and a large panel, respectively, in two cases before the current WES analysis. One recommendation for trial participation from the WES MTB was biomarker independent. One variant would have been detectable with the larger Oncomine panel. ESCAT tiers were all assigned retrospectively.

ap, accredited pipeline; CDK, cyclin-dependent kinas; DKFZ, German Cancer Research Center; DKTK, German Cancer Consortium; ERBB, erythroblastic oncogene B; ESCAT, European Society for Medical Oncology Scale for Clinical Actionability of molecular Targets; FGFR, fibroblast growth factor receptor; LoE, level of evidence; MTB, molecular tumor board; NCT, National Center for Tumor Diseases; OCA, Oncomine Comprehensive Assay V2; OFA, Oncomine Focus Assay; PARP, poly (ADP-ribose) polymerase; PD-(L)1, programmed death-(ligand) 1; PIK3CA, phosphatidylinositol-4,5-bisphosphate 3-kinase catalytic subunit alpha; SOS1, Son of Sevenless Homolog 1; TMB, tumor mutational burden; WES, whole-exome sequencing.

a DKFZ/NCT/DKTK MASTER LoEs were assigned retrospectively.

**Figure 3:**
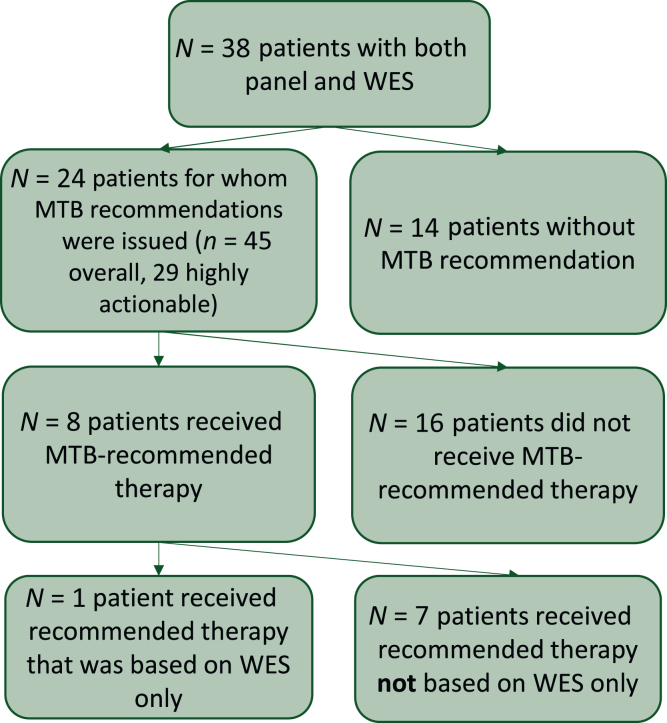
**CONSORT diagram.** Receipt of therapy recommendations (overall and actionable) and the implementation of these recommendations per patient in our cohort. CONSORT, Consolidated Standards of Reporting Trials; MTB, molecular tumor board; WES, whole-exome sequencing.

No potentially actionable alterations were detected in 14 patients (37%, Figure 3) and no highly actionable mutations with a clinical LoE (up to m2C) or a feasible biomarker-based study enrollment were detected in 19 patients (50%, including the 14 patients without highly actionable alterations). In the other cases (*n* = 11, 29%), all clinical-level alterations were (additionally or exclusively) detected in panel sequencing. Of the eight alterations that were exclusively detected by panel sequencing, six were amplifications that we could not detect with our WES pipeline, and the remaining two were judged to be germline alterations.

### Implementation of MTB recommendations

Overall, 8 out of 24 patients with therapy recommendations received one of the biomarker-dependent MTB-recommended therapies (Figure 3 and Table 2). In one of these patients (patient 2), the implemented MTB-recommended therapy was a checkpoint inhibitor based on a high mutational burden detected by our WES only. In one additional case, one patient (patient 5) received the recommended checkpoint inhibitor with a preclinical LoE biomarker independently in conjunction with lenvatinib, and two patients received a checkpoint inhibitor based on high TMB detected by previous sequencing [one WGS (patient 11), one large panel (patient 26)]. Table 2 summarizes all implemented molecularly guided therapies and their outcome.

**Table 2:** Implemented recommendations

Patient	Cancer	Recommended	Biomarker	Implemented	Within clinical trial (yes/no)	Best response (as reported)	PFS (months)
2	Mammary carcinoma (lobular) with brain metastases	Pembrolizumab	TMB-high (28.2 mut/Mb)	Pembrolizumab	No	SD	2
6	Large cell neuroendocrine carcinoma of the lung, no metastases	PARP inhibitor	CHEK2 variant (p.T367Mfs∗15)	Olaparib	No	SD	7
8	Urothelial carcinoma	Erdafitinib	FGFR3 variant (p.S249C)	Erdafitinib	No	PD	NR
9	High-grade serous ovarian cancer with peritoneal, pelvic and perihepatic metastases	Lenvatinib, alternatively ponatinib, erdafitinib, pemigatinib	FGFR1 amplification	Lenvatinib	No	SD	2
11	Lung adenocarcinoma with brain metastases	PD-L1 antibody (monotherapy due to Crohn disease)	TMB-high (1102 SNVs, 21 indels according to previous analysis)	Nivolumab	No	SD	10
26	TTF1+ poorly differentiated adenocarcinoma of the lung with brain and lung metastases	Checkpoint inhibitor within IMP 150 regimen (carboplatin/paclitaxel/bevacizumab/atezolizumab)	TMB-high (previous analysis with neoplus assay)	Impower-150 regimen	No	PR	27+, ongoing
35	High-grade serous ovarian cancer with peritoneal and lymph node metastases	Pembrolizumab + lenvatinib	Amplification in FGFR1, FGF3, FGFR19	Pembrolizumab/lenvatinib	No	NR	4
36	Squamous-cell carcinoma of the thymus with liver metastases	Platinum-based chemotherapy	BAP1 variant (p.C91G)	Carboplatin/paclitaxel	No	PR	8

PARP, poly (ADP-ribose) polymerase; PD, progressive disease; PD-L1, programmed death-ligand 1; PFS, progression-free survival; PR, partial response; SD, stable disease; TMB, tumor mutational burden. NR, not reported.

Regarding the reasons for non-implementation of the recommended therapies, as discussed above, 14 patients had not received any therapy recommendations due to a lack of actionable biomarkers. Among patients who received therapy recommendations, three patients progressed and deteriorated too much before any recommended therapies could be initiated. Three patients were lost to follow-up before treatment initiation. One patient declined the recommended treatment. Two of the patients are currently still on first-line therapy. One patient participated in another study than the MTB-recommended one. In only one case did the health insurance refuse to cover the costs, which correlates well with the fact that almost only therapies with clinical evidence levels were applied for. In the other cases, the reason for therapy non-implementation was not available.

## Discussion

In this study, we report the results of a real-life cohort of 38 patients with advanced, mostly common cancers from a single institution, who were discussed in our MTB and received both panel sequencing and WES. We only observed a limited direct benefit in terms of therapy recommendations by carrying out WES analyses in addition to panel analysis. That was particularly the case if only recommendations with a clinical LoE were considered, and even more so if implementation was taken into account. Moreover, as already alluded to in the ‘Introduction’ section, WES or even broader analyses such as WGS are known to be more ‛costly’ than panels in terms of actual required reagents, hardware and software as well as patient material, but also in terms of time and expertise required for interpreting as well as reporting the data to treating physicians and patients. The latter is especially critical with respect to variants of unknown significance and their (non-)actionability.^14^^,^^16^

Our results are broadly in line with the findings in previous studies reporting similar comparisons in other settings, which also found a distinct, but limited benefit of additional WES or WGS^15^^,^^17^, ^18^, ^19^ (also see case vignettes of the Hartwig Foundation^104^). Importantly, Kerle et al.^15^ very recently reported a similar implementation rate of WES-only-based therapy recommendations (2 out of 10) in a study that was conducted in a large precision oncology program (DKFZ/NCT/DKTK MASTER) focused on rare cancer entities and younger patients and not in a ‛routine’ MTB as in our study. The observed benefit in our study was mostly due to the detection of complex biomarkers such as high TMB, microsatellite instability or HRD, which are challenging to detect in smaller panels such as the ones we routinely used in this study, but can be derived from larger panels, as also illustrated by one case in our study where a high TMB was determined using a large panel.^14^^,^^105^

Direct benefit, i.e. an increase in the number of implementable therapies, from precision oncology approaches in general is thought to be substantial for patients with rare cancer entities,^4^^,^^5^^,^^9^^,^^15^ where more extensive biomarker analysis including WES or even WGS may serve to discover experimental therapies for patients without standard therapies. In addition, patients with common cancer entities where many biomarker-driven therapies have been established already may also benefit from panel diagnostics early on.^9^ In this case, panel diagnostics may enable the selection of the most promising sequential or concurrent combination therapies. In our MTB, however, patients with common cancers were usually heavily pretreated and potential benefit resulted mostly from TMB and HRD analysis, also for common cancers where such molecular patterns are not routinely assessed. Thus, a molecular analysis workflow that permits the detection or at least the estimation of such molecular patterns, possibly also within a large gene panel, should be implemented in all MTBs, as it may yield useful therapeutic targets relatively frequently. As a rate-limiting step in broad molecular diagnostics often is the analysis rather than the wet-lab part, and as sequencing costs are dropping, one could also envisage a workflow where wet-lab WES with a standard virtual panel is initially carried out, which could then easily be extended to further panels if no adequate targets are detected. However, the larger the (actual or virtual) panel that is used for a routine MTB, the smaller the difference in overall ‛cost’ will be compared with WES.

The question of whether and when it might also be helpful to issue therapy recommendations on the basis of preclinical evidence is still open, as usually only systemic therapy recommendations that are based on clinical evidence levels are reimbursed after prior case-based authorization by public health insurance and thus recommendations with lower LoEs are often not implemented.^9^^,^^106^ While the largest benefit is usually observed for the LoEs/tiers with the highest clinical evidence (e.g. Belcaid et al.^107^), in a recent study, ESCAT tier I alterations leading to recommendations in an MTB were still associated with the largest clinical benefit, but the authors point out that also tier II and III alterations led to promising results, i.e. successful recruitment of patients into clinical trials or improved survival.^108^ In our study, only therapies with clinical LoEs were applied for and administered to the patients, at the discretion of the treating physicians.

Although more difficult to assess, indirect, but nevertheless crucial, patient benefit from precision oncology approaches may also result from knowledge generation regarding the molecular setup and potential drivers and resistance mechanisms—again, particularly for rare and thus often not well-characterized cancer entities.^4^^,^^5^^,^^9^^,^^15^ In addition, as new therapy options might arise over time, immediate availability of extended molecular analyses for certain patients might enable fast implementation of these treatments once they become available. An added benefit from WES/WGS is the automatically generated germline data.

Although larger targeted panels may also help to identify novel driver mutations,^109^^,^^110^ WES or WGS, nowadays often complemented by whole transcriptome analysis,^4^^,^^8^ is the more informative analysis regarding knowledge generation with a view to enhancing our understanding of cancer evolution and resistance, and to shed light on as yet unknown molecular drivers of cancer and potential new therapeutic targets.^14^ Such endeavors, however, are ultimately only helpful if the omics results are systematically evaluated and recurrent alterations are translated into clinical trials or at least systematic case series, which is not achievable in most real-world MTBs due to the associated high personnel, time and funding requirements. Thus, according to our experience, a standard MTB with a clear focus on immediate patient benefit can usually rely on comprehensive, well-established targeted sequencing panels on most occasions. In contrast, and in line with the current national and international strategies, it is likely that broader diagnostics will be concentrated in specialized centers such as Comprehensive Cancer Centers with adequate resources, increasing speed and quality and decreasing cost and time requirements via high throughput, and sparing smaller institutions the need to establish complex, costly and most likely underutilized pipelines or even departments.

As new actionable drivers continue to be found, and well-established driver mutations are periodically incorporated into targeted sequencing panels (e.g. https://www.mskcc.org/msk-impact), we strongly recommend a regular re-assessment of the selection of the panel(s) that is/are utilized to inform the MTB discussions. Moreover, as recent studies have shown that targeted RNA sequencing or transcriptomics analysis may also yield potentially actionable targets to a similar or even higher extent, RNA panels or the RNA proportion of targeted sequencing panels is likely to evolve into a more important parameter guiding cancer therapy selection in the near future.^6^^,^^19^^,^^110^^,^^111^ Therefore, it is also vital to incorporate this modality optimally into MTB procedures.

### Limitations

Our cohort was relatively small, with a broad real-world composition of rare and common tumor entities and pretreatments. Larger studies and/or studies with more homogeneous cohorts would be required to determine the potential benefit for individual patient groups. This is particularly true for indirect patient benefit through discovery of novel targets, which we could not assess in the present study. Molecular analysis was also heterogeneous, as the employed panels and pipelines changed over time and with respect to patient age. In addition, in the majority of cases, the recommended therapies were not implemented. Thus, we cannot determine the clinical benefit of the WES-dependent recommended therapies overall. Of note, many of these recommendations were based on complex biomarkers that are associated with a relatively high therapy success rate across many cancer types, namely HRD^112^ and high TMB,^103^ and/or mutations associated with these patterns.

In general, there is still a large evidence gap regarding the optimal implementation strategy for precision medicine, with a view to maximizing gains of lifetime and quality of life, but at the same time maintaining a tolerable level of cost and expert workload. Several European countries are currently implementing dedicated precision medicine programs, especially in the field of oncology, with a view to enabling access to precision medicine for every citizen. Prominent examples are the German ‛Model Project Genome Sequencing’ (https://www.bfarm.de/EN/BfArM/Tasks/Model-Project-Genome-Sequencing/_node.html), the French 2025 Genomic Medicine Initiative (https://pfmg2025.fr/en/) or the European PCM4EU (https://health.ec.europa.eu/non-communicable-diseases/cancer/europes-beating-cancer-plan-eu4health-financed-projects/projects/pcm4eu_en), and precision oncology trials like the Dutch Drug Rediscovery Protocol (DRUP, NCT02925234) trials or the recently initiated RATIONALE trial (NCT06855134) focusing on rare cancers are ongoing and will contribute to answering the existing open questions regarding the optimal setup of precision oncology programs and their benefit for patients. Moreover, ongoing Europe-wide efforts to increase high-level cancer care to all European citizens, such as JaNE2 (https://jane-2.eu/), CCI4EU (https://cci4eu.eu/) or EUnetCCC (https://eunetccc2025.eu/), also promote access to and further development of expertise in precision oncology. As with other medical areas such as surgery, special training and expertise and standardized workflows are required especially for broader (non-panel) molecular diagnostics and the interpretation of their results.

### Conclusions

In summary, in our real-life MTB cohort, using established panels of low to moderate sizes and an in-house WES pipeline, we observed a limited direct benefit in terms of therapy recommendations of WES over panel sequencing. To a substantial extent, this benefit was due to the detection of complex molecular patterns. Overall, this suggests that in the current situation, real-life all-comer MTBs can achieve good direct patient benefit with limited financial and temporal cost through larger gene panels that also enable analysis of genomic patterns in the absence of WES. These findings should be confirmed in future, larger studies, also encompassing patient outcomes, before firm conclusions regarding the optimal extent of molecular diagnostics for cancer patients in an all-comer MTB can be drawn.
