# Supplementary material for: Clinical benefit of additional whole-exome sequencing over panel sequencing in an all-comer real-world molecular tumor board
Source: ESMO Open. 2025 Nov 25;10(12):105894. doi: 10.1016/j.esmoop.2025.105894 (PMC12689208; doi:10.1016/j.esmoop.2025.105894)
Supplement: Supplementary Figures and Tables [file mmc1.docx]

**Supplement “Clinical benefit of additional whole exome sequencing over panel sequencing in an all-comer real-world molecular tumor board.”**

**Supplementary Table 1: Panel comparison**

| **panel/pipeline** | **OFA** | **OCAv3** | **OCCRA** | **accr. pipeline** |
| --- | --- | --- | --- | --- |
| **hotspot mut** |  |  | ABL1 |  |
|  |  |  | ABL2 |  |
|  |  |  |  |  |
|  |  |  | ACVR1 |  |
|  | AKT1 | AKT1 | AKT1 |  |
|  |  | AKT2 |  |  |
|  |  | AKT3 |  |  |
|  | ALK | ALK | ALK |  |
|  | AR | AR |  |  |
|  |  | ARAF |  |  |
|  |  |  | ASXL1 |  |
|  |  |  | ASXL2 |  |
|  |  | AXL |  |  |
|  | BRAF | BRAF | BRAF |  |
|  |  | BTK |  |  |
|  |  |  | CALR |  |
|  |  | CBL | CBL |  |
|  |  | CCND1 | CCND1 |  |
|  |  |  | CCND3 |  |
|  |  |  | CCR5 |  |
|  | CDK4 | CDK4 | CDK4 |  |
|  |  | CDK6 |  |  |
|  |  | CHEK2 |  |  |
|  |  |  | CIC |  |
|  |  |  | CREBBP |  |
|  |  |  | CRLF2 |  |
|  |  | CSF1R | CSF1R |  |
|  |  |  | CSF3R |  |
|  | CTNNB1 | CTNNB1 | CTNNB1 |  |
|  |  |  | DAXX |  |
|  | DDR2 | DDR2 |  |  |
|  |  |  | DNMT3A |  |
|  | EGFR | EGFR | EGFR |  |
|  |  |  | EP300 |  |
|  | ERBB2 | ERBB2 | ERBB2 |  |
|  | ERBB3 | ERBB3 | ERBB3 |  |
|  | ERBB4 | ERBB4 | ERBB4 |  |
|  |  | ERCC2 | ESR1 |  |
|  | ESR1 | ESR1 | EZH2 |  |
|  |  | EZH2 |  |  |
|  |  |  | FASLG |  |
|  |  |  | FBXW7 |  |
|  |  | FGFR1 | FGFR1 |  |
|  | FGFR2 | FGFR2 | FGFR2 |  |
|  | FGFR3 | FGFR3 | FGFR3 |  |
|  |  | FGFR4 |  |  |
|  |  | FLT3 | FLT3 |  |
|  |  | FOXL2 |  |  |
|  |  | GATA2 | GATA2 |  |
|  | GNA11 | GNA11 | GNA11 |  |
|  | GNAQ | GNAQ | GNAQ |  |
|  |  | GNAS |  |  |
|  |  | H3F3A | H3F3A |  |
|  |  |  | HDAC9 |  |
|  |  | HIST1H3B | HIST1H3B |  |
|  |  | HNF1A |  |  |
|  | HRAS | HRAS | HRAS |  |
|  | IDH1 | IDH1 | IDH1 |  |
|  | IDH2 | IDH2 | IDH2 |  |
|  |  |  | IL7R |  |
|  | JAK1 | JAK1 | JAK1 |  |
|  | JAK2 | JAK2 | JAK2 |  |
|  | JAK3 | JAK3 | JAK3 |  |
|  |  |  | KDM4C |  |
|  |  | KDR | KDR |  |
|  | KIT | KIT | KIT |  |
|  |  | KNSTRN |  |  |
|  |  | KRAS | KRAS |  |
|  |  | MAGOH |  |  |
|  | MAP2K1 | MAP2K1 | MAP2K1 |  |
|  |  | MAP2K2 | MAP2K2 |  |
|  |  | MAP2K4 |  |  |
|  |  | MAPK1 |  |  |
|  |  | MAX |  |  |
|  |  | MDM4 |  |  |
|  |  | MED12 |  |  |
|  | MET | MET | MET |  |
|  |  |  | MPL |  |
|  |  |  | MSH6 |  |
|  |  | MTOR | MTOR |  |
|  | MYC | MYC | MYC |  |
|  | MYCN | MYCN | MYCN |  |
|  |  | MYD88 |  |  |
|  |  |  | NCOR2 |  |
|  |  | NFE2L2 |  |  |
|  |  |  | NOTCH1 |  |
|  |  |  | NPM1 |  |
|  |  | NRAS | NRAS |  |
|  |  |  | NT5C2 |  |
|  |  | NTRK1 |  |  |
|  |  | NTRK2 |  |  |
|  |  | NTRK3 |  |  |
|  |  |  | PAX5 |  |
|  | PDFGRA | PDGFRA | PDGFRA |  |
|  |  | PDGFRB | PDGFRB |  |
|  | PIK3CA | PIK3CA | PIK3CA |  |
|  |  | PIK3CB |  |  |
|  |  |  | PIK3R1 |  |
|  |  |  | PPM1D |  |
|  |  | PPP2R1A |  |  |
|  |  | PTPN11 | PTPN11 |  |
|  |  | RAC1 |  |  |
|  |  | RAF1 | RAF1 |  |
|  |  | RET | RET |  |
|  |  | RHEB |  |  |
|  |  | RHOA | RHOA |  |
|  |  | ROS1 |  |  |
|  |  |  | SETBP1 |  |
|  |  |  | SETD2 |  |
|  |  | SF3B1 |  |  |
|  |  |  | SH2B3 |  |
|  |  |  | SH2D1A |  |
|  |  | SMAD4 |  |  |
|  |  | SMO | SMO |  |
|  |  | SPOP |  |  |
|  |  | SRC |  |  |
|  |  | STAT3 | STAT3 |  |
|  |  |  | STAT5B |  |
|  |  | TERT | TERT |  |
|  |  | TOP1 |  |  |
|  |  |  | TPMT |  |
|  |  | U2AF1 |  |  |
|  |  |  | USP7 |  |
|  |  | XPO1 |  |  |
|  |  |  | ZMYM3 |  |
|  |  |  |  |  |
| **CNV** |  |  | ABL2 |  |
|  |  | AKT1 |  |  |
|  |  | AKT2 |  |  |
|  |  | AKT3 |  |  |
|  | ALK | ALK | ALK |  |
|  | AR | AR |  |  |
|  |  | AXL |  |  |
|  | BRAF | BRAF | BRAF |  |
|  | CCND1 | CCND1 | CCND1 |  |
|  |  | CCND2 |  |  |
|  |  | CCND3 |  |  |
|  |  | CCNE1 |  |  |
|  |  | CDK2 |  |  |
|  | CDK4 | CDK4 | CDK4 |  |
|  | CDK6 | CDK6 | CDK6 |  |
|  | EGFR | EGFR | EGFR |  |
|  | ERBB2 | ERBB2 | ERBB2 |  |
|  |  |  | ERBB3 |  |
|  |  | ESR1 |  |  |
|  |  | FGF19 |  |  |
|  |  | FGF3 |  |  |
|  | FGFR1 | FGFR1 | FGFR1 |  |
|  | FGFR2 | FGFR2 | FGFR2 |  |
|  | FGFR3 | FGFR3 | FGFR3 |  |
|  | FGFR4 | FGFR4 | FGFR4 |  |
|  |  | FLT3 |  |  |
|  |  |  | GLI1 |  |
|  |  |  | GLI2 |  |
|  |  | IGF1R | IGF1R |  |
|  |  |  | JAK1 |  |
|  |  |  | JAK2 |  |
|  |  |  | JAK3 |  |
|  | KIT | KIT | KIT |  |
|  | KRAS | KRAS | KRAS |  |
|  |  | MDM2 | MDM2 |  |
|  |  | MDM4 | MDM4 |  |
|  | MET | MET | MET |  |
|  | MYC | MYC | MYC |  |
|  | MYCN | MYCL |  |  |
|  |  | MYCN | MYCN |  |
|  |  | NTRK1 |  |  |
|  |  | NTRK2 |  |  |
|  |  | NTRK3 |  |  |
|  | PDGFRA | PDGFRA | PDGFRA |  |
|  |  | PDGFRB |  |  |
|  | PIK3CA | PIK3CA | PIK3CA |  |
|  |  | PIK3CB |  |  |
|  |  | PPARG |  |  |
|  |  | RICTOR |  |  |
|  |  | TERT |  |  |
| **fusions** | ABL1 |  | ABL1 |  |
|  |  |  | ABL2 |  |
|  |  |  | AFF3 |  |
|  | AKT1 |  |  |  |
|  |  | AKT2 |  |  |
|  | ALK | ALK | ALK |  |
|  |  | AR |  |  |
|  | AXL | AXL |  |  |
|  |  |  | BCL11B |  |
|  |  |  | BCOR |  |
|  |  |  | BCR |  |
|  | BRAF | BRAF | BRAF |  |
|  |  | BRCA1 |  |  |
|  |  | BRCA2 |  |  |
|  |  |  | CAMTA1 |  |
|  |  |  | CCND1 |  |
|  |  | CDKN2A |  |  |
|  |  |  | CIC |  |
|  |  |  | CREBBP |  |
|  |  |  | CRLF2 |  |
|  |  |  | CSF1R |  |
|  |  |  | DUSP22 |  |
|  |  | EGFR |  |  |
|  | ERBB2 | ERBB2 | EGFR |  |
|  |  | ERBB4 |  |  |
|  | ERG | ERG |  |  |
|  |  | ESR1 |  |  |
|  | ETV1 | ETV1 |  |  |
|  | ETV5 | ETV4 |  |  |
|  |  | ETV5 |  |  |
|  |  |  | ETV6 |  |
|  |  |  | EWSR1 |  |
|  | FGFR1 | FGFR1 | FGFR1 |  |
|  | FGFR2 | FGFR2 | FGFR2 |  |
|  | FGFR3 | FGFR3 | FGFR3 |  |
|  |  | FGR |  |  |
|  |  | FLT3 | FLT3 |  |
|  |  |  | FOSB |  |
|  |  |  | FUS |  |
|  |  |  | GLI1 |  |
|  |  |  | GLIS2 |  |
|  |  |  | HMGA2 |  |
|  |  | JAK2 | JAK2 |  |
|  |  |  | KAT6A |  |
|  |  |  | KMT2A |  |
|  |  |  | KMT2B |  |
|  |  |  | KMT2C |  |
|  |  |  | KMT2D |  |
|  |  | KRAS |  |  |
|  |  |  | LMO2 |  |
|  |  |  | MAML2 |  |
|  |  |  | MAN2B1 |  |
|  |  | MDM4 |  |  |
|  |  |  | MECOM |  |
|  |  |  | MEF2D |  |
|  |  | MET | MET |  |
|  |  |  | MKL1 |  |
|  |  |  | MLLT10 |  |
|  |  |  | MN1 |  |
|  |  | MYB | MYB |  |
|  |  | MYBL1 | MYBL1 |  |
|  |  |  | MYH11 |  |
|  |  |  | MYH9 |  |
|  |  |  | NCOA2 |  |
|  |  |  | NCOR1 |  |
|  |  | NF1 |  |  |
|  |  | NOTCH1 | NOTCH1 |  |
|  |  |  | NOTCH2 |  |
|  |  | NOTCH4 | NOTCH4 |  |
|  |  |  | NPM1 |  |
|  |  |  | NR4A3 |  |
|  |  | NRG1 |  |  |
|  | NTRK1 | NTRK1 | NTRK1 |  |
|  | NTRK2 | NTRK2 | NTRK2 |  |
|  | NTRK3 | NTRK3 | NTRK3 |  |
|  |  |  | NUP214 |  |
|  |  |  | NUP98 |  |
|  |  | NUTM1 | NUTM1 |  |
|  |  |  | NUTM2B |  |
|  | PAR1 |  |  |  |
|  |  |  | PAX3 |  |
|  |  |  | PAX5 |  |
|  |  |  | PAX7 |  |
|  |  |  | PDGFB |  |
|  | PDGFRA | PDGFRA | PDGFRA |  |
|  |  | PDGFRB | PDGFRB |  |
|  |  | PIK3CA |  |  |
|  |  |  | PLAG1 |  |
|  | PPARG | PPARG |  |  |
|  |  | PRKACA |  |  |
|  |  | PRKACB |  |  |
|  |  | PTEN |  |  |
|  |  | RAD51B |  |  |
|  |  | RAF1 | RAF1 |  |
|  |  |  | RANBP17 |  |
|  |  |  | RARA |  |
|  |  | RB1 |  |  |
|  |  |  | RECK |  |
|  |  | RELA | RELA |  |
|  | RET | RET | RET |  |
|  | ROS1 | ROS1 | ROS1 |  |
|  |  | RSPO2 |  |  |
|  |  | RSPO3 |  |  |
|  |  |  | RUNX1 |  |
|  |  |  | SS18 |  |
|  |  |  | SSBP2 |  |
|  |  |  | STAG2 |  |
|  |  |  | STAT6 |  |
|  |  |  | TAL1 |  |
|  |  |  | TCF3 |  |
|  |  | TERT |  |  |
|  |  |  | TFE3 |  |
|  |  |  | TP63 |  |
|  |  |  | TSLP |  |
|  |  |  | TSPAN4 |  |
|  |  |  | UBTF |  |
|  |  |  | USP6 |  |
|  |  |  | WHSC1 |  |
|  |  |  | YAP1 |  |
|  |  |  | ZMYND11 | |
|  |  |  | ZNF384 |  |
| **exon skipping** | EGFR |  |  |  |
|  | MET |  |  |  |
|  |  |  |  |  |
| **full exon coverage** | |  |  | A1CF |
|  |  |  |  | ABCB1 |
|  |  |  |  | ABL1 |
|  |  |  |  | ABL2 |
|  |  |  |  | ABRAXAS1 |
|  |  |  |  | ACSM2B |
|  |  |  |  | ACVR1 |
|  |  |  |  | ACVR1B |
|  |  |  |  | ACVR2A |
|  |  |  |  | ADAM18 |
|  |  |  |  | ADAMTS12 |
|  |  |  |  | ADAMTS2 |
|  |  |  |  | AIP |
|  |  |  |  | AKT1 |
|  |  |  |  | AKT2 |
|  |  |  |  | AKT3 |
|  |  |  |  | ALK |
|  |  |  |  | AMER1 |
|  |  |  |  | ANO4 |
|  |  |  | APC | APC |
|  |  |  |  | AR |
|  |  |  |  | ARAF |
|  |  |  |  | ARHGAP35 |
|  |  | ARID1A | ARID1A | ARID1A |
|  |  |  | ARID1B | ARID1B |
|  |  |  |  | ARID2 |
|  |  |  |  | ARID5B |
|  |  |  |  | ASXL1 |
|  |  |  |  | ASXL2 |
|  |  | ATM |  | ATM |
|  |  |  |  | ATP1A1 |
|  |  | ATR |  | ATR |
|  |  | ATRX | ATRX | ATRX |
|  |  |  |  | AURKA |
|  |  |  |  | AURKB |
|  |  |  |  | AURKC |
|  |  |  |  | AXIN1 |
|  |  |  |  | AXIN2 |
|  |  |  |  | AXL |
|  |  |  |  | B2M |
|  |  | BAP1 |  | BAP1 |
|  |  |  |  | BARD1 |
|  |  |  |  | BCL10 |
|  |  |  |  | BCL2 |
|  |  |  |  | BCL2L12 |
|  |  |  |  | BCL6 |
|  |  |  |  | BCOR |
|  |  |  |  | BCR |
|  |  |  |  | BIRC3 |
|  |  |  |  | BLM |
|  |  |  |  | BMP5 |
|  |  |  |  | BMPR1A |
|  |  |  |  | BMPR2 |
|  |  |  |  | BRAF |
|  |  | BRCA1 |  | BRCA1 |
|  |  | BRCA2 |  | BRCA2 |
|  |  |  |  | BRINP3 |
|  |  |  |  | BRIP1 |
|  |  |  |  | BTK |
|  |  |  |  | BUB1B |
|  |  |  |  | C6 |
|  |  |  |  | C8A |
|  |  |  |  | C8B |
|  |  |  |  | CACNA1D |
|  |  |  |  | CALR |
|  |  |  |  | CANX |
|  |  |  |  | CARD11 |
|  |  |  |  | CASP8 |
|  |  |  |  | CASR |
|  |  |  |  | CBFB |
|  |  |  |  | CBL |
|  |  |  |  | CCND1 |
|  |  |  |  | CCND2 |
|  |  |  |  | CCND3 |
|  |  |  |  | CCNE1 |
|  |  |  |  | CD163 |
|  |  |  |  | CD274 |
|  |  |  |  | CD276 |
|  |  |  |  | CD79B |
|  |  |  |  | CDC73 |
|  |  |  |  | CDH1 |
|  |  |  |  | CDH10 |
|  |  |  |  | CDK12 |
|  |  |  |  | CDK2 |
|  |  |  |  | CDK4 |
|  |  |  |  | CDK6 |
|  |  | CDK12 |  |  |
|  |  |  |  | CDKN1A |
|  |  | CDKN1B |  | CDKN1B |
|  |  |  |  | CDKN1C |
|  |  | CDKN2A | CDKN2A | CDKN2A |
|  |  | CDKN2B | CDKN2B | CDKN2B |
|  |  |  |  | CDKN2C |
|  |  |  | CEBPA | CEBPA |
|  |  |  |  | CHD4 |
|  |  |  | CHD7 |  |
|  |  | CHEK1 |  | CHEK1 |
|  |  |  |  | CHEK2 |
|  |  |  |  | CIC |
|  |  |  |  | CIITA |
|  |  |  |  | CNTN6 |
|  |  |  |  | CNTNAP4 |
|  |  |  |  | CNTNAP5 |
|  |  |  |  | COL11A1 |
|  |  |  |  | COL3A1 |
|  |  | CREBBP |  | CREBBP |
|  |  |  | CRLF1 |  |
|  |  |  |  | CRLF2 |
|  |  |  |  | CSF1R |
|  |  |  |  | CSF3R |
|  |  |  |  | CSMD3 |
|  |  |  |  | CTCF |
|  |  |  |  | CTLA4 |
|  |  |  |  | CTNNA1 |
|  |  |  |  | CTNNB1 |
|  |  |  |  | CTNND2 |
|  |  |  |  | CUL1 |
|  |  |  |  | CUL3 |
|  |  |  |  | CUL4A |
|  |  |  |  | CUL4B |
|  |  |  |  | CXCR4 |
|  |  |  |  | CYLD |
|  |  |  |  | CYP2C9 |
|  |  |  |  | CYP2D6 |
|  |  |  |  | CYSLTR2 |
|  |  |  |  | DAXX |
|  |  |  |  | DCAF4L2 |
|  |  |  |  | DCDC1 |
|  |  |  |  | DDB2 |
|  |  |  |  | DDR1 |
|  |  |  |  | DDR2 |
|  |  |  | DDX3X | DDX3X |
|  |  |  |  | DEK |
|  |  |  |  | DGCR8 |
|  |  |  | DICER1 | DICER1 |
|  |  |  |  | DLST |
|  |  |  |  | DNMT3A |
|  |  |  |  | DOCK3 |
|  |  |  |  | DOCK6 |
|  |  |  |  | DPYD |
|  |  |  |  | DROSHA |
|  |  |  |  | DSC1 |
|  |  |  |  | DSC2 |
|  |  |  |  | DSC3 |
|  |  |  |  | DSG2 |
|  |  |  |  | DSP |
|  |  |  |  | DUSP22 |
|  |  |  |  | E2F1 |
|  |  |  | EBF1 |  |
|  |  |  |  | ECT2L |
|  |  |  | EED | EED |
|  |  |  |  | EGFR |
|  |  |  |  | EGLN1 |
|  |  |  |  | EGLN2 |
|  |  |  |  | EIF1AX |
|  |  |  |  | ELF3 |
|  |  |  |  | EMSY |
|  |  |  |  | ENO1 |
|  |  |  |  | EP300 |
|  |  |  |  | EPAS1 |
|  |  |  |  | EPCAM |
|  |  |  |  | EPHA2 |
|  |  |  |  | EPOR |
|  |  |  |  | ERAP1 |
|  |  |  |  | ERAP2 |
|  |  |  |  | ERBB2 |
|  |  |  |  | ERBB3 |
|  |  |  |  | ERBB4 |
|  |  |  |  | ERCC2 |
|  |  |  |  | ERCC3 |
|  |  |  |  | ERCC4 |
|  |  |  |  | ERCC5 |
|  |  |  |  | ERG |
|  |  |  |  | ERRFI1 |
|  |  |  |  | ESR1 |
|  |  |  |  | ETNK1 |
|  |  |  |  | ETV1 |
|  |  |  |  | ETV4 |
|  |  |  |  | ETV5 |
|  |  |  |  | ETV6 |
|  |  |  |  | EXT1 |
|  |  |  |  | EXT2 |
|  |  |  |  | EZH2 |
|  |  |  |  | FAM135B |
|  |  | FANCA |  | FANCA |
|  |  |  |  | FANCB |
|  |  |  |  | FANCC |
|  |  | FANCD2 |  | FANCD2 |
|  |  |  |  | FANCE |
|  |  |  |  | FANCF |
|  |  |  |  | FANCG |
|  |  | FANCI |  | FANCI |
|  |  |  |  | FANCL |
|  |  |  |  | FANCM |
|  |  |  | FAS | FAS |
|  |  |  |  | FAT1 |
|  |  |  |  | FBN1 |
|  |  | FBXW7 |  | FBXW7 |
|  |  |  |  | FGF19 |
|  |  |  |  | FGF23 |
|  |  |  |  | FGF3 |
|  |  |  |  | FGF4 |
|  |  |  |  | FGF7 |
|  |  |  |  | FGF9 |
|  |  |  |  | FGFR1 |
|  |  |  |  | FGFR2 |
|  |  |  |  | FGFR3 |
|  |  |  |  | FGFR4 |
|  |  |  |  | FGR |
|  |  |  |  | FH |
|  |  |  |  | FLCN |
|  |  |  |  | FLI1 |
|  |  |  |  | FLT3 |
|  |  |  |  | FLT4 |
|  |  |  |  | FOXA1 |
|  |  |  |  | FOXL2 |
|  |  |  |  | FOXO1 |
|  |  |  |  | FOXP1 |
|  |  |  |  | FUBP1 |
|  |  |  |  | FYN |
|  |  |  | GATA1 | GATA1 |
|  |  |  |  | GATA2 |
|  |  |  | GATA3 | GATA3 |
|  |  |  |  | GLI1 |
|  |  |  |  | GLI3 |
|  |  |  |  | GNA11 |
|  |  |  | GNA13 | GNA13 |
|  |  |  |  | GNAQ |
|  |  |  |  | GNAS |
|  |  |  |  | GPC3 |
|  |  |  |  | GPR158 |
|  |  |  |  | GPS2 |
|  |  |  |  | GREM1 |
|  |  |  |  | GRID2 |
|  |  |  |  | H1-4 |
|  |  |  |  | H2BC5 |
|  |  |  |  | H3-3A |
|  |  |  |  | H3-3B |
|  |  |  |  | H3C2 |
|  |  |  |  | HCN1 |
|  |  |  |  | HDAC2 |
|  |  |  |  | HDAC9 |
|  |  |  |  | HIF1A |
|  |  |  |  | HLA-A |
|  |  |  |  | HLA-B |
|  |  |  |  | HLA-C |
|  |  |  |  | HNF1A |
|  |  |  |  | HOXB13 |
|  |  |  |  | HRAS |
|  |  |  | ID3 | ID3 |
|  |  |  |  | IDH1 |
|  |  |  |  | IDH2 |
|  |  |  |  | IGF1R |
|  |  |  |  | IKBKB |
|  |  |  | IKZF1 | IKZF1 |
|  |  |  |  | IL3 |
|  |  |  |  | IL6ST |
|  |  |  |  | IL7R |
|  |  |  |  | INPP4B |
|  |  |  |  | IRF4 |
|  |  |  |  | IRS4 |
|  |  |  |  | JAK1 |
|  |  |  |  | JAK2 |
|  |  |  |  | JAK3 |
|  |  |  |  | KCND2 |
|  |  |  |  | KCNH7 |
|  |  |  |  | KCNJ5 |
|  |  |  |  | KDM5C |
|  |  |  | KDM6A | KDM6A |
|  |  |  |  | KDR |
|  |  |  |  | KEAP1 |
|  |  |  |  | KEL |
|  |  |  |  | KIT |
|  |  |  |  | KLF2 |
|  |  |  |  | KLF4 |
|  |  |  |  | KLF5 |
|  |  |  |  | KLHL13 |
|  |  |  |  | KMT2A |
|  |  |  |  | KMT2B |
|  |  |  |  | KMT2C |
|  |  |  | KMT2D | KMT2D |
|  |  |  |  | KNSTRN |
|  |  |  |  | KRAS |
|  |  |  |  | KRTAP2-1 |
|  |  |  |  | KRTAP6-2 |
|  |  |  |  | LARP4B |
|  |  |  |  | LATS1 |
|  |  |  |  | LATS2 |
|  |  |  |  | LDLR |
|  |  |  |  | LMO2 |
|  |  |  |  | LRRC7 |
|  |  |  |  | LZTR1 |
|  |  |  |  | MAF |
|  |  |  |  | MAFB |
|  |  |  |  | MAGOH |
|  |  |  |  | MALT1 |
|  |  |  |  | MAP2K1 |
|  |  |  |  | MAP2K2 |
|  |  |  |  | MAP2K4 |
|  |  |  |  | MAP2K7 |
|  |  |  |  | MAP3K1 |
|  |  |  |  | MAP3K4 |
|  |  |  |  | MAPK1 |
|  |  |  |  | MAPK8 |
|  |  |  |  | MARCO |
|  |  |  |  | MAX |
|  |  |  |  | MC1R |
|  |  |  |  | MCL1 |
|  |  |  |  | MDH2 |
|  |  |  |  | MDM2 |
|  |  |  |  | MDM4 |
|  |  |  |  | MECOM |
|  |  |  |  | MED12 |
|  |  |  |  | MEF2B |
|  |  |  |  | MEN1 |
|  |  |  |  | MET |
|  |  |  |  | MITF |
|  |  | MLH1 |  | MLH1 |
|  |  |  |  | MLH3 |
|  |  |  |  | MLLT10 |
|  |  |  |  | MPL |
|  |  | MRE11 |  | MRE11 |
|  |  | MSH2 |  | MSH2 |
|  |  |  |  | MSH3 |
|  |  | MSH6 |  | MSH6 |
|  |  |  |  | MSMB |
|  |  |  |  | MTAP |
|  |  |  |  | MTOR |
|  |  |  |  | MTUS2 |
|  |  |  |  | MUTYH |
|  |  |  |  | MYB |
|  |  |  |  | MYBL1 |
|  |  |  |  | MYC |
|  |  |  |  | MYCL |
|  |  |  |  | MYCN |
|  |  |  |  | MYD88 |
|  |  |  | MYOD1 | MYOD1 |
|  |  | NBN |  | NBN |
|  |  |  |  | NCOR1 |
|  |  | NF1 | NF1 | NF1 |
|  |  | NF2 | NF2 | NF2 |
|  |  |  |  | NFE2L2 |
|  |  |  |  | NLRC5 |
|  |  |  |  | NOL4 |
|  |  | NOTCH1 |  | NOTCH1 |
|  |  | NOTCH2 |  | NOTCH2 |
|  |  | NOTCH3 |  | NOTCH3 |
|  |  |  |  | NOTCH4 |
|  |  |  |  | NPM1 |
|  |  |  |  | NRAS |
|  |  |  |  | NRG1 |
|  |  |  |  | NRXN1 |
|  |  |  |  | NSD2 |
|  |  |  |  | NT5C2 |
|  |  |  |  | NTHL1 |
|  |  |  |  | NTRK1 |
|  |  |  |  | NTRK2 |
|  |  |  |  | NTRK3 |
|  |  |  |  | NUP93 |
|  |  |  |  | NUTM1 |
|  |  |  |  | NYAP2 |
|  |  |  |  | ODAD2 |
|  |  |  |  | OR10G8 |
|  |  |  |  | OR2G6 |
|  |  |  |  | OR2L13 |
|  |  |  |  | OR2L2 |
|  |  |  |  | OR2L8 |
|  |  |  |  | OR2M3 |
|  |  |  |  | OR2T3 |
|  |  |  |  | OR2T33 |
|  |  |  |  | OR2T4 |
|  |  |  |  | OR2W3 |
|  |  |  |  | OR4A15 |
|  |  |  |  | OR4C15 |
|  |  |  |  | OR4C6 |
|  |  |  |  | OR4M1 |
|  |  |  |  | OR4M2 |
|  |  |  |  | OR5D18 |
|  |  |  |  | OR5F1 |
|  |  |  |  | OR5L1 |
|  |  |  |  | OR5L2 |
|  |  |  |  | OR6F1 |
|  |  |  |  | OR8H2 |
|  |  |  |  | OR8I2 |
|  |  |  |  | OR8U1 |
|  |  |  |  | ORC4 |
|  |  |  |  | PAK5 |
|  |  | PALB2 |  | PALB2 |
|  |  |  |  | PARP1 |
|  |  |  |  | PARP2 |
|  |  |  |  | PARP3 |
|  |  |  |  | PARP4 |
|  |  |  |  | PAX5 |
|  |  |  |  | PCBP1 |
|  |  |  |  | PCDH17 |
|  |  |  |  | PDCD1 |
|  |  |  |  | PDCD1LG2 |
|  |  |  |  | PDE1A |
|  |  |  |  | PDE1C |
|  |  |  |  | PDGFB |
|  |  |  |  | PDGFRA |
|  |  |  |  | PDGFRB |
|  |  |  |  | PDIA3 |
|  |  |  |  | PGD |
|  |  |  | PHF6 | PHF6 |
|  |  |  |  | PHOX2B |
|  |  |  |  | PIK3C2B |
|  |  |  |  | PIK3CA |
|  |  |  |  | PIK3CB |
|  |  |  |  | PIK3CD |
|  |  |  |  | PIK3CG |
|  |  | PIK3R1 |  | PIK3R1 |
|  |  |  |  | PIK3R2 |
|  |  |  |  | PIM1 |
|  |  |  |  | PLCG1 |
|  |  |  |  | PLXDC2 |
|  |  |  |  | PMS1 |
|  |  | PMS2 |  | PMS2 |
|  |  |  |  | POLD1 |
|  |  | POLE |  | POLE |
|  |  |  |  | POM121L12 |
|  |  |  |  | POT1 |
|  |  |  |  | PPARG |
|  |  |  |  | PPFIA2 |
|  |  |  |  | PPM1D |
|  |  |  |  | PPP2R1A |
|  |  |  |  | PPP2R2A |
|  |  |  |  | PPP6C |
|  |  |  |  | PRDM1 |
|  |  |  |  | PRDM9 |
|  |  |  |  | PRKACA |
|  |  |  |  | PRKACB |
|  |  |  |  | PRKAR1A |
|  |  |  | PRPS1 |  |
|  |  |  |  | PRSS1 |
|  |  |  | PSMB5 |  |
|  |  |  |  | PSMB8 |
|  |  |  |  | PSMB9 |
|  |  |  |  | PSMB10 |
|  |  | PTCH1 | PTCH1 | PTCH1 |
|  |  | PTEN | PTEN | PTEN |
|  |  |  |  | PTPN11 |
|  |  |  |  | PTPRD |
|  |  |  |  | PTPRT |
|  |  |  |  | PXDNL |
|  |  |  |  | RAC1 |
|  |  | RAD50 |  | RAD50 |
|  |  | RAD51 |  | RAD51 |
|  |  | RAD51B |  | RAD51B |
|  |  | RAD51C |  | RAD51C |
|  |  | RAD51D |  | RAD51D |
|  |  |  |  | RAD52 |
|  |  |  |  | RAD54L |
|  |  |  |  | RAF1 |
|  |  |  |  | RARA |
|  |  |  |  | RASA1 |
|  |  |  |  | RASA2 |
|  |  | RB1 | RB1 | RB1 |
|  |  |  |  | RBM10 |
|  |  |  |  | RBM15 |
|  |  |  |  | RBP3 |
|  |  |  |  | RECQL4 |
|  |  |  |  | REG1A |
|  |  |  |  | REG1B |
|  |  |  |  | REG3A |
|  |  |  |  | REG3G |
|  |  |  |  | RELA |
|  |  |  |  | RELN |
|  |  |  |  | RET |
|  |  |  |  | RGS7 |
|  |  |  |  | RHBDF2 |
|  |  |  |  | RHEB |
|  |  |  |  | RHOA |
|  |  |  |  | RICTOR |
|  |  |  |  | RIT1 |
|  |  |  |  | RNASEH2A |
|  |  |  |  | RNASEH2B |
|  |  |  |  | RNASEH2C |
|  |  | RNF43 |  | RNF43 |
|  |  |  |  | ROS1 |
|  |  |  |  | RPA1 |
|  |  |  |  | RPL10 |
|  |  |  |  | RPL22 |
|  |  |  |  | RPL5 |
|  |  |  |  | RPS6KB1 |
|  |  |  |  | RPTN |
|  |  |  |  | RPTOR |
|  |  |  |  | RSPO2 |
|  |  |  |  | RSPO3 |
|  |  |  |  | RUNDC3B |
|  |  |  | RUNX1 | RUNX1 |
|  |  |  |  | SDHA |
|  |  |  |  | SDHAF2 |
|  |  |  |  | SDHB |
|  |  |  |  | SDHC |
|  |  |  |  | SDHD |
|  |  |  |  | SEC23B |
|  |  |  |  | SETBP1 |
|  |  | SETD2 |  | SETD2 |
|  |  |  |  | SF3B1 |
|  |  |  |  | SH2B3 |
|  |  |  |  | SH3RF2 |
|  |  |  |  | SIX1 |
|  |  |  |  | SIX2 |
|  |  |  |  | SLC15A2 |
|  |  |  |  | SLC8A1 |
|  |  |  |  | SLCO1B3 |
|  |  | SLX4 |  | SLX4 |
|  |  |  |  | SMAD2 |
|  |  |  |  | SMAD4 |
|  |  | SMARCA4 | SMARCA4 | SMARCA4 |
|  |  | SMARCB1 | SMARCB1 | SMARCB1 |
|  |  |  |  | SMARCE1 |
|  |  |  |  | SMC1A |
|  |  |  |  | SMO |
|  |  |  |  | SNCAIP |
|  |  |  |  | SOCS1 |
|  |  |  | SOCS2 |  |
|  |  |  |  | SOS1 |
|  |  |  |  | SOX2 |
|  |  |  |  | SOX9 |
|  |  |  |  | SPC24 |
|  |  |  |  | SPEN |
|  |  |  |  | SPINK1 |
|  |  |  |  | SPOP |
|  |  |  |  | SRC |
|  |  |  |  | SRSF2 |
|  |  |  |  | STAG2 |
|  |  |  |  | STAT1 |
|  |  |  |  | STAT3 |
|  |  |  |  | STAT5B |
|  |  |  |  | STAT6 |
|  |  | STK11 |  | STK11 |
|  |  |  | SUFU | SUFU |
|  |  |  | SUZ12 | SUZ12 |
|  |  |  |  | SYT10 |
|  |  |  |  | SYT16 |
|  |  |  |  | TAF1 |
|  |  |  |  | TAL1 |
|  |  |  |  | TAP1 |
|  |  |  |  | TAP2 |
|  |  |  |  | TAPBP |
|  |  |  |  | TBX3 |
|  |  |  | TCF3 | TCF3 |
|  |  |  |  | TCF7L2 |
|  |  |  |  | TCL1A |
|  |  |  |  | TCL1B |
|  |  |  |  | TERT |
|  |  |  | TET2 | TET2 |
|  |  |  |  | TGFBR1 |
|  |  |  |  | TGFBR2 |
|  |  |  |  | TINF2 |
|  |  |  |  | TLX1 |
|  |  |  |  | TLX3 |
|  |  |  |  | TMEM127 |
|  |  |  |  | TMEM132D |
|  |  |  |  | TNFAIP3 |
|  |  |  |  | TNFRSF14 |
|  |  |  |  | TOP1 |
|  |  |  |  | TOP2A |
|  |  | TP53 | TP53 | TP53 |
|  |  |  |  | TP63 |
|  |  |  |  | TPMT |
|  |  |  |  | TPP2 |
|  |  |  |  | TPTE |
|  |  |  |  | TRHDE |
|  |  |  |  | TRIM48 |
|  |  |  |  | TRIM51 |
|  |  |  |  | TRRAP |
|  |  | TSC1 | TSC1 | TSC1 |
|  |  | TSC2 | TSC2 | TSC2 |
|  |  |  |  | TSHR |
|  |  |  |  | TYK2 |
|  |  |  |  | U2AF1 |
|  |  |  |  | UGT1A1 |
|  |  |  |  | USP8 |
|  |  |  |  | USP9X |
|  |  |  |  | VHL |
|  |  |  |  | WAS |
|  |  |  | WHSC1 |  |
|  |  |  |  | WRAP53 |
|  |  |  |  | WRN |
|  |  |  | WT1 | WT1 |
|  |  |  | XIAP |  |
|  |  |  |  | XPA |
|  |  |  |  | XPC |
|  |  |  |  | XPO1 |
|  |  |  |  | XRCC2 |
|  |  |  |  | XRCC3 |
|  |  |  |  | YAP1 |
|  |  |  |  | YES1 |
|  |  |  |  | ZBTB20 |
|  |  |  |  | ZFHX3 |
|  |  |  |  | ZIM3 |
|  |  |  |  | ZMYM3 |
|  |  |  |  | ZNF217 |
|  |  |  |  | ZNF429 |
|  |  |  |  | ZNF479 |
|  |  |  |  | ZNF536 |
|  |  |  |  | ZRSR2 |
| **gene expression** |  |  | BCL2 |  |
|  |  |  | BCL6 |  |
|  |  |  | FGFR1 |  |
|  |  |  | FGFR4 |  |
|  |  |  | IGF1R |  |
|  |  |  | MET |  |
|  |  |  | MYC |  |
|  |  |  | MYCN |  |
|  |  |  | TOP2A |  |

**Supplementary Table 2: Basic characteristics of the patient cohort**

| **pat** | **age at diagnosis** | **sex** | **disease entity** | **previous genetic analysis results** | **PD-L1 status** | **prior systemic therapies (either (neo-)adjuvant or palliative** |
| --- | --- | --- | --- | --- | --- | --- |
| pat1 | 52 | m | rectal carcinoma (additional papillary renal cell carcinoma) | KRAS variant, MSS, no BRAF variant | TPS 0%, ICS 3%, CPS 20% | FOLFOXIRI, FOLFOX6, FOLFIRI/ bevacizumab |
| pat2 | 57 | f | lobular mammary carcinoma, HR+/HER2- | none | unknown | Letrozole/ribociclib/denosumab; [intrathecal MTX] |
| pat3 | 59 | m | prostate carcinoma, Gleason 9 | no BRCA variant, pathogenic FGFR4 variant | unknown | cyproterone acetate/triptorelin, docetaxel, abiraterone, enzalutamide, docetaxel re-challenge, PSMA ligand therapy, cabazitaxel |
| pat4 | 67 | f | mammary carcinoma | PIK3CA wt | unknown | Docetaxel/cyclophosphamide, tamoxifen, letrozole/ribociclib , liposomal doxorubicin, carboplatin/gemcitabine, eribulin |
| pat5 | 79 | m | intrahepatic cholangiocellular carcinoma | none | unknown | none |
| pat6 | 38 | f | large cell neuroendocrine carcinoma, most likely originating in the lung | none | unknown | Carboplatin/etoposide (without/with pegfilgrastim) |
| pat7 | 64 | m | prostate carcinoma | none | unknown | docetaxel |
| pat8 | 62 | m | urothelial carcinoma | none | n.a. | Gemcitabin/cisplatin, vinflunin, pembrolizumab, vinflunin re-challenge |
| pat9 | 58 | f | high-grade serous ovarian carcinoma | none | TPS: 0% ICS: 0% CPS: 0 | Carboplatin/paclitaxel, carboplatin/liposomal doxorubicin/bevacizumab, topotecan |
| pat10 | 46 | f | HNSCC (?) | CCND1 amplification | unknown | unknown |
| pat11 | 35 | m | lung adenocarcinoma | RET variant | probably positive, no further information | Platinum derivative /pemetrexed, selpercatinib |
| pat12 | 50 | m | large cell carcinoma of the lung with partial neuroendocrine differentiation, TTF1- | none | unknown | unknown |
| pat13 | 66 | f | adenocarcinoma, most likely of the ovary | none | unknown | Carboplatin/paclitaxel/bevacizumab, carboplatin/liposomal doxorubicin, topotecan |
| pat14 | 42 | m | bone sarcoma | none | unknown | unknown |
| pat15 | 61 | f | high-grade serous ovarian carcinoma | none | unknown | Carboplatin/paclitaxel/bevacizumab , niraparib, liposomal doxorubicin/carboplatin |
| pat16 | 61 | f | high-grade serous ovarian carcinoma | none | unknown | Carboplatin/Paclitaxel, liposomal Doxorubicin/Bevacizumab/Atezolizumab within the AGO2-29 trial |
| pat17 | 35 | f | pregnancy-associated mammary carcinoma, HR+/HER2- | BRCA1/2 wt, PIK3CA wt | unknown | Epirubicin/cyclophosphamide/paclitaxel, tamoxifen, palbociclib/letrozole/goserelin , exemestane/goserelin/everolimus , bevacizumab/capecitabine, liposomal doxorubicin, (nab-paclitaxel planned) |
| pat18 | 61 | f | mammary carcinoma, HR+ HER2- | MTOR Exon 39 variant p.E1799K, ESR1 Exon 9 variant p.D538G, ESR1-AKAP12 fusion, PIK3CA WT, BRCA 1/2 WT (germ line, somatic) | unknown | Letrozole, fulvestrant/denosumab, palbociclib(3 weeks)/letrozole/denosumab, everolimus/exemestane/denosumab, bevacizumab/capecitabine/zoledronic Acid, nab-paclitaxel/zoledronic acid |
| pat19 | 36 | m | rectal carcinoma | MSS, KRAS p.G12S, NRAS wt, BRAF wt, HER2- | unknown | Capecitabine (within the neoadjuvant radiochemotherapy), capecitabine, FOLFIRI, FOLFIRI/bevacizumab |
| pat20 | 33 | m | adenocarcinoma of the esophagogastral junction | MSS, HER2- | TPS < 1 %, IC 5 %, CPS 6 | FLOT, FOLFIRI/ramucirumab |
| pat21 | 42 | f | poorly differentiated urothelial carcinoma, merging into an undifferentiated spindle cell-like carcinoma | Faktor-V-Leiden variant | unknown/n.a. | Gemcitabin/cisplatin, gemcitabin/carboplatin/pembrolizumab |
| pat22 | 59 | f | low-grade serous ovarian carcinoma | BRCA1/2-Status unknown, prior mammary carcinoma | unknown | Carboplatin/paclitaxel/bevacizumab, carboplatin/gemcitabin/niraparib, carboplatin, letrozole, trametinib, paclitaxel, fulvestrant/palbociclib |
| pat23 | 47 | f | colon carcinoma | MSS | unknown | FOLFOX, FOLFIRI/panitumumab |
| pat24 | 56 | m | rectal carcinoma | MSS, POLE c.1A>T germline variant | unknown/n.a. | Capecitabine (within radiochemotherapy), FOLFIRINOX, FOLFIRI/bevacizumab |
| pat25 | 74 | m | thyroid carcinoma (additionally follicular lymphoma) | FGFR4 and NRAS variants | unknown | radioiodine therapy, rituximab-bendamustin |
| pat26 | 52 | f | poorly differentiated lung adenocarcinoma, TTF+ | TMB 10,32 Mut/Mb | TPS 0 %, 5 % IC | Cisplatin/vinorelbine, osimertinib |
| pat27 | 61 | f | high-grade serous ovarian carcinoma, on the basis of a borderline tumor | BRCA 1/2 wt (germline) | unknown | participation in the AGO-Ovar/DUO-O-trial: carboplatin/paclitaxel/bevacizumab + durvalumab/placebo + olaparib/placebo, bevacizumab maintenance; carboplatin; participation in the AGO Ovar 2.29 trial: paclitaxel + bevacizumab +/- atezolizumab (running, current therapy therefore unclear due to blinding) |
| pat28 | 44 | f | cecum carcinoma | none | unknown/n.a. | FOLFOXIRI |
| pat29 | 32 | f | mammary carcinoma, HR+/HER2-, later triple-negative | PIK3CA missense exon 21 variant p.G1049R (class 4), PIK3CA amplification; BRCA 1/2 WT (germline) | liver metastasis: TPS 5%, IC 1%, CPS 6 | Epirubicin/cyclophosphamide/paclitaxel, tamoxifen/goserelin, palbociclib/letrozole/placebo + giredestrant/placebo within the PERSEVERA trial, denosumab, participation in the ATRACTIB trial: atezolizumab/paclitaxel/bevacizumab |
| pat30 | 64 | f | lung adenocarcinoma, TTF1+ | KRAS p.G12C (OCA) | TPS 20 % | Carboplatin/pemetrexed/pembrolizumab/zoledronic acid, pemetrexed/pembrolizumab, sotorasib (compassionate use)/zoledronic acid |
| pat31 | 64 | m | large cell carcinoma of the lung with partial neuroendocrine differentiation; TTF1- | none prior to OCA | TPS 0 % | Carboplatin/nab-paclitaxel/atezolizumab |
| pat32 | 37 | f | neuroendocrine tumor, most likely originating in the biliopancreatic or upper gastrointestinal tract | none | unknown | unknown |
| pat33 | 43 | m | cholangiocellular carcinoma | none | unknown | Capecitabine, gemcitabine/cisplatin/durvalumab |
| pat34 | 22 | f | small cell neuroendocrine carcinoma, DD small cell transformed teratoma | none | unknown | Carboplatin/etoposide, atezolizumab |
| pat35 | 53 | f | high-grade serous ovarian carcinoma, additionally endometroid adenocarcinoma, follicular lymphoma | MSS | unknown | Carboplatin/paclitaxel, carboplatin/gemcitabine/bevacizumab, carboplatin/bevacizumab |
| pat36 | 54 | m | squamous cell carcinoma of the thymus | none | unknown | Carboplatin/paclitaxel (after OCA decision and before WES Pembrolizumab) |
| pat37 | 57 | m | lung adenocarcinoma, TTF1+ | none | TPS 0 %, IC 0 %, CPS 0 % | Carboplatin/pemetrexed/pembrolizumab, pemetrexed/embrolizumab |
| pat38 | 26 | m | rectal carcinoma | MSS, RAS/BRAF- negative - APC p.E1309*, TP53 p.R196*, ASXL2 p.P396S, WT1 p.D469V; DPD: heterozygous for 2A variant | PD-L1 negative | FOLFIRI, FOLFIRI/cetuximab |

**
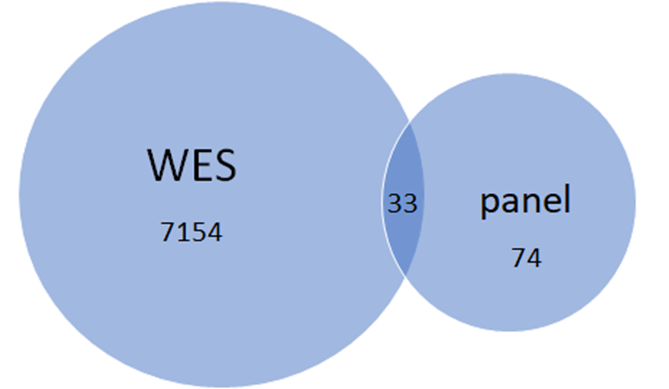
**

**Supplementary Figure 1: Venn diagram of individual alterations.** About one third of the alterations detected via panel sequencing were also identified via WES. Fusions and CNVs (n=4 and n=19, respectively) detected by panel sequencing cannot be detected with the WES pipeline. Germline variants are not called in our WES pipeline, whereas they are detected, but not defined as germline alterations, in panel sequencing. Complex molecular biomarkers such as high TMB and HRD are not included in this diagram, although they are detected by WES and can lead to recommendations.
